# Behavioral models of impulsivity in relation to ADHD: Translation between clinical and preclinical studies

**DOI:** 10.1016/j.cpr.2006.01.001

**Published:** 2006-08

**Authors:** Catharine A. Winstanley, Dawn M. Eagle, Trevor W. Robbins

**Affiliations:** Department of Experimental Psychology, University of Cambridge, CB2 3EB, UK

**Keywords:** ADHD, Impulsivity, Frontal cortex, Inhibition, Serotonin, Dopamine

## Abstract

Impulsivity, broadly defined as action without foresight, is a component of numerous psychiatric illnesses including attention deficit/hyperactivity disorder (ADHD), mania and substance abuse. In order to investigate the mechanisms underpinning impulsive behavior, the nature of impulsivity itself needs to be defined in operational terms that can be used as the basis for empirical investigation. Due to the range of behaviors that the term impulsivity describes, it has been suggested that impulsivity is not a unitary construct, but encompasses a variety of related phenomena that may differ in their biological basis. Through fractionating impulsivity into these component parts, it has proved possible to devise different behavioral paradigms to measure various aspects of impulsivity in both humans and laboratory animals. This review describes and evaluates some of the current behavioral models of impulsivity developed for use with rodents based on human neuropsychological tests, focusing on the five-choice serial reaction time task, the stop-signal reaction time task and delay-discounting paradigms. Furthermore, the contributions made by preclinical studies using such methodology to improve our understanding of the neural and neurochemical basis of impulsivity and ADHD are discussed, with particular reference to the involvement of both the serotonergic and dopaminergic systems, and frontostriatal circuitry.

## 1 Introduction

Impulsivity is a characteristic of human behavior that can be both beneficial and detrimental to our everyday lives. For example, the ability to act on impulse may allow us to seize a valuable opportunity, or to make a disastrous decision that we then live to regret. Impulsivity can be viewed as a dimension of normal personality (Eysenck & Eysenck, 1977), but high levels of impulsivity are associated with psychiatric disorders such as ADHD, mania, substance abuse and personality disorders, indicating that this personality trait can be maladaptive (DSM IV, 1994). It has been suggested that impulsivity is not a unitary construct (Evenden, 1999; Moeller, Barratt, Dougherty, Schmitz, & Swann, 2001), and that increases in different aspects of impulsivity may represent different subtypes of ADHD (Nigg, 2003; Sonuga-Barke, 2002).

The aim of this review is to discuss current thought regarding the nature of impulse control, and to evaluate the advancements that have been made in improving our understanding of the neural and neurochemical basis of impulsive behavior. Integral to furthering progress in this field has been the development of behavioral paradigms designed to measure impulsivity in laboratory animals, based on human neuropsychological tests. Data from clinical and preclinical studies are compared and areas of agreement and diversity underlined, with particular reference to multi-process models of both ADHD and impulse control. Future research directions are also suggested which may help resolve some of the issues and inconsistencies present in the literature.

## 2 The multi-faceted nature of impulsivity

Although impulsivity can be broadly defined as action without foresight, researchers and psychiatrists alike have struggled to formulate a single definition of impulsivity that captures every aspect of behavior encompassed by this concept. A commonly used technique in clinical psychology to measure and identify different aspects of behavior is through the use of self-report questionnaires. A number of such questionnaires have been designed in order to try to quantify and qualify impulsivity in both normal adult and patient populations, such as the Barratt Impulsiveness scale (Barratt, 1994) and the I7 (Eysenck, 1993), but measures of impulsivity are also included in other more general assessments of personality such as the Karolinska Scales of Personality, and the Tridimensional Personality Questionnaire (Cloninger, 1987).

Factor analysis of self-report questionnaires suggests that impulsive behavior consists of several independent dimensions, although there is considerable variation as to the precise definition of these constituent parts (see Evenden, 1999 for review). However, common themes include decreased inhibitory control, intolerance of delay to rewards and quick decision-making due to lack of consideration, as well as more universal deficits such as poor attentional ability. Therefore, one definition of impulsivity which seems particularly appropriate is that “impulsivity encompasses a range of actions which are poorly conceived, prematurely expressed, unduly risky or inappropriate to the situation and that often result in undesirable consequences” (Daruna & Barnes, 1993).

## 3 Measuring impulsivity in the laboratory

Given the diversity of behavior that the term impulsivity appears to cover, it has been suggested that impulsivity is not a unitary construct at all, but is used to classify a variety of phenomena that may have independent underlying biological mechanisms (Evenden, 1999). Through focusing on different aspects of impulsive behavior, it has proved possible to devise a variety of behavioral paradigms to measure impulsivity in both human and non-human subjects. These can be broadly divided into two categories: those measuring impulsive choice or impulsive decision-making, and those measuring impulsive action or motoric impulsivity.

## 4 Measuring impulsive action

Impulsive action can be broadly defined as the inability to withhold from making a response. Within the framework of behavioral neuroscience and cognitive psychology, impulse control has been described as an active inhibitory mechanism which modulates the internally or externally driven pre-potent desire for primary reinforcers such as food, sex or other highly desirable rewards. This inhibitory control mechanism may provide the substrate by which rapid conditioned responses and reflexes are transiently suppressed, so that slower cognitive mechanisms can guide behavior. There is an extensive and complex literature on the neural and neurochemical basis of different types of behavioral inhibition (e.g. see Nigg, 2000 for review), the in-depth discussion of which would fall outside the remit of this review, and only the work most relevant to the concept of impulsivity will be considered here.

## 5 Go/no-go and stop-signal reaction time tasks

Two of the most common tests used to study inhibitory processes in both clinical and non-clinical populations are the go/no-go and stop-signal reaction time (SSRT) tasks (see Band & Van Boxtel, 1999 for recent review). In a typical go/no-go paradigm, following the initiation of a trial, the subject learns to make a particular response when cued to do so by the “go” signal (e.g. to touch the stimulus on a screen or to press a key). However, on a subset of trials, the “no-go” signal is presented, either concurrently with the “go” signal or prior to it, whereupon the subject must withhold from making the pre-potent response. The design of the stop-task is very similar, with the exception that the “no-go” or “stop” signal occurs after the presentation of the “go” signal. The closer the stop signal is in time to the moment of responding, the harder it is for the subject to inhibit their behavior.

Go/no-go (Harrison, Everitt, & Robbins, 1999), stop-change (Feola, de Wit, & Richards, 2000) and, more recently, SSRT paradigms (Eagle & Robbins, 2003a,b) have been developed for use with rats with some success. In the stop-task, rats are trained to respond rapidly and accurately on first one and then the other of two levers. Correct execution of this response sequence results in the delivery of reward. The average time taken to perform this response is calculated and referred to as the mean go reaction time (mRT). On 20% of the trials in a session, a stop-signal is presented in the form of a tone after the rat has responded on the first lever but prior to responding on the second lever. Subjects must then inhibit their response on the second lever in order to receive a reward. Failure to refrain from making this pre-potent response is punished not only by the absence of reward but also by a 5-s timeout, during which the subject cannot engage in the task to earn reward.

In order to assess the ability of a subject to inhibit behavior, the stop-signal reaction time (SSRT) is calculated, which is essentially an estimation of the time taken to inhibit the go response. The SSRT cannot be measured directly, as it is impossible to measure the latency to a response that does not occur. Therefore it is derived using the “horse-race” model developed by Logan and Cowan (1984) which uses the mRT to estimate the SSRT (see Logan, 1994 for further details concerning the “horse-race” model and its application to the stop-task). SSRTs estimated by this method are close to 200 ms for human adult subjects (Logan & Cowan, 1984), but may exceed 400 ms in young children (Schachar & Logan, 1990) and the elderly (Kramer, Humphrey, Larish, Logan, & Strayer, 1994), as well as in adults classified as ‘impulsive’ (Logan, Schachar, & Tannock, 1997) and hyperactive children (Rubia, Oosterlaan, Sergeant, Brandeis, & v Leeuwen, 1998; Schachar & Logan, 1990). For rats, SSRTs have been estimated to be around 300 ms using a conventional SSRT paradigm (Eagle & Robbins, 2003a,b), and between 100 and 150 ms using a ‘stop-change’ version of the task (Feola et al., 2000).

## 6 The five-choice serial reaction time task

Many behavioral tasks invoke inhibitory processes at some stage and can be useful for measuring aspects of impulsivity. An example of a task used in rats which was not designed with the sole purpose of measuring impulsive behavior but which does require an aspect of behavioral inhibition, is the five-choice serial reaction time task (5CSRT; Carli, Robbins, Evenden, & Everitt, 1983). This task was developed as a test of sustained and divided attention for rats based on the continuous performance task (CPT) used to monitor attentional function in humans (Rosvold, Mirsky, Sarason, Bransome, & Beck, 1956; Wilkinson, 1963).

During the 5CSRT, the animal is required to make a response in one of five response apertures only when a stimulus light located therein is illuminated. Subsequent to beginning a trial and prior to illumination of a stimulus light, there is a 5-s inter-trial interval (ITI) during which the animal must withhold from responding in the apertures. Any responses made during this time are described as premature responses and are punished. These premature responses provide another way of measuring motoric impulsivity and are potentially analogous to “false alarm” errors made in the CPT. One of the advantages of using a task such as the 5CSRT which measures multiple aspects of performance (attention, impulsivity, motivation, speed of responding etc.) is that it is possible to dissociate the effects of various manipulations of the central nervous system (CNS) on different types of behavior.

## 7 Measuring impulsive choice

Impulsivity is also evident in the making of impulsive decisions or choices as well as in impulsive actions. It has been argued that all impulsivity involves an impulsive action of some sort, in that it is necessary to perform an action in order to select a response alternative. However the key difference conceptually is that, unlike for measurements of impulsive action, there is no “pre-potent” response that is primed and then forcibly inhibited. Furthermore, impulsive choice reflects, to a greater degree, decision-making processes rather than motoric inhibition.

## 8 Delay-discounting paradigms

**Fig. 1:**
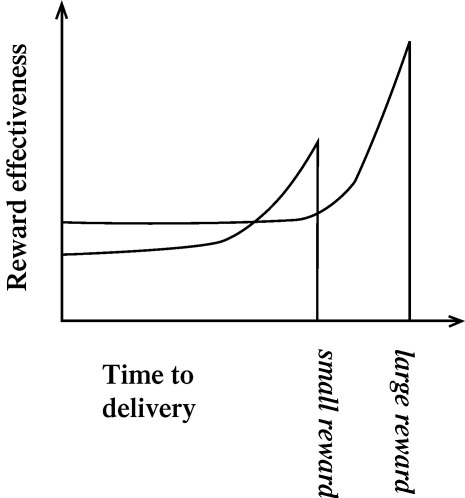
Impulsive choice and preference reversal as predicted by hyperbolic discounting functions (adapted from Ainslie, 1975). Hyperbolic discounting models predict that a larger but more delayed reward will be valued more highly when the choice is made far in advance of its delivery (towards the left of the graph). However, as time advances, and the difference between the delay to the small reward is judged as being considerably less than the delay to the large reward, preference switches so that the small reward is deemed to be more valuable.

One of the most successfully utilised measurements of impulsive choice is intolerance to delay-of-gratification, or delay-discounting, which is the function by which a reward is subjectively devalued by a delay to its delivery. All measurements of delay-discounting, whether operant behavioral tasks or questionnaire-based measures, essentially pose the question of whether a smaller but more immediate reward is worth more or less than a larger but delayed reward see (Ainslie, 1975; Logue, 1988). Under these circumstances, impulsive choice is defined as the selection of the smaller immediate reward. The vast majority of behavioral data strongly indicate that the function relating the subjective value of reward to the magnitude and delay to the delivery of that reward is hyperbolic (see Ainslie, 1975 for review). Such functions predict impulsive choice in that there is a point in time at which the smaller reward is preferred more than the larger but more delayed reward, and this tendency to choose the impulsive option decreases as the delay to the large reward shortens (see Fig. 1).

Devising a sensitive operant delay-discounting task for clinical research has proved difficult due to the essentially limited temporal duration of laboratory measures of behavior, in that human subjects tend not to find it sufficiently aversive to delay gratification over such short periods of time. Nevertheless, there has been one very recent report utilising such an operant delay-discounting procedure (Pietras, Cherek, Lane, Tcheremissine, & Steinberg, 2003) based on the adjusting amount task designed for use with rats (see below for details). The majority of data regarding delay-discounting in the clinical psychology literature, however, has been obtained using questionnaires. On the contrary, a number of different delay-discounting tasks have been developed for use with laboratory animals. In all delay-discounting paradigms, the subject essentially chooses between responding on one lever which leads to a small reward and another which leads to a large but delayed reward. Such tasks can be divided into “systematic” tasks, where the experimenter varies the delay to different sized reinforcers and then measures the number of choices made of the large reward at different delays (e.g. Evenden & Ryan, 1996; Mobini, Chiang, Ho, Bradshaw, & Szabadi, 2000; Wogar, Bradshaw, & Szabadi, 1993), or “adjusting” tasks in which the behavior of the subject determines the delays sampled (Mazur, 1987; Richards, Mitchell, de Wit, & Seiden, 1997), all of which have made valuable contributions to the literature.

## 9 Impulsivity and ADHD

ADHD is essentially categorised by inattentive, hyperactive and impulsive behavior. Both the attentional and impulse control deficits seen in ADHD patients can be illustrated by their performance of the CPT. ADHD subjects have slower and more variable reaction times, and make more errors of omission indicative of poor attentional ability (e.g. (Epstein et al., 2003). In addition, those with ADHD make more errors of commission, demonstrating reduced behavioral inhibition. Indeed, ADHD patients show elevated levels of impulsivity as measured by a variety of tasks (see e.g. Solanto, 1998, 2002 for reviews). Focusing on the SSRT task, children with ADHD are slower to inhibit their responses than normal children, as indicated by increases in their SSRT (Nigg, 1999; Purvis & Tannock, 2000; Schachar & Logan, 1990; Schachar, Tannock, Marriott, & Logan, 1995), and similarly fail to cancel their “go” response on the “no-go” trials in go/no-go tasks.

It is this inability to inhibit a pre-potent response that is thought to be one of the fundamental deficits which characterises ADHD. However, some studies indicate that children with ADHD are impaired in their performance of other aspects of the stop-task. For example, there is often a slowing of reaction time on the go trials, as indicated by increases in the mRT, as well as on the stop-trials, which may be indicative of a general processing-speed impairment rather than a specific inhibitory deficit (Oosterlaan, Logan, & Sergeant, 1998; Overtoom et al., 2002; Tannock, 1998). Furthermore, it has also been reported that ADHD children make more errors of commission on a choice reaction time version of the task (i.e. choose the wrong response on the go trials), and omit more trials (i.e. do not respond at all: Bedard et al., 2003; Kuntsi, Oosterlaan, & Stevenson, 2001; Overtoom et al., 2003; Tannock, Schachar, Carr, Chajczyk, & Logan, 1989). Such increases in omissions may have serious consequences for the analysis of the data, as the “horse-race” model used to calculate the SSRT cannot account for such changes in response strategy (Eagle & Robbins, 2003a,b; Solanto et al., 2001; Tannock et al., 1989).

The interpretation of the stop-task performance of ADHD children is therefore far from straightforward, and it has been suggested that differences between the outcomes of various studies using diverse populations of ADHD patients could reflect different subtypes of the disorder. Furthermore, increased SSRT is not a unique behavioral symptom confined to those patients with ADHD, but has been observed in other patient groups including those with damage to the frontal lobes and basal ganglia (Aron, Fletcher, Bullmore, Sahakian, & Robbins, 2003; Rieger, Gauggel, & Burmeister, 2003). Further investigation into such patterns of deficits may provide valuable insight into the processes critically involved in inhibitory control, hence enhancing our understanding of the deficiencies in impulsive control observed in ADHD.

ADHD patients also choose more impulsively in delay-discounting tasks, preferring the smaller but more immediate rewards to the larger more delayed rewards (Schweitzer & Sulzer-Azaroff, 1995; Solanto et al., 2001; Sonuga-Barke, Taylor, Sembi, & Smith, 1992; Sonuga-Barke, Williams, Hall, & Saxton, 1996). However, if the selection of a smaller immediate reward does not reduce the total length of the time the subject spends engaged in the experimental task, it has been reported that ADHD patients are able to wait for rewards. It has been suggested that this pattern of impulsive choice is indicative of enhanced motivation to escape or avoid delay, and that the inattentive, overactive and impulsive behaviors in which ADHD patients engage are functional expressions of delay-aversion (Sonuga-Barke, 2002, 2003). However, it would appear there is a double dissociation between preference for delayed rewards and behavioral disinhibition, in that ADHD patients can choose to wait for large rewards under certain circumstances, even when such a choice involves ongoing inhibitory control (Sonuga-Barke et al., 1992, 1996). Furthermore, the level of inhibitory control ADHD patients show, as typified by the SSRT, and their preference for large delayed rewards, as assessed using delay-discounting paradigms, are *not* correlated (Solanto et al., 2001). Nevertheless, these two performance measures together have proved to be highly diagnostic, identifying over 90% of patients, confirming that poor inhibitory control and elevated impulsive choice are core symptoms of ADHD.

Such data have lead to the hypothesis that there may be two subtypes of ADHD which lead to the generation of ADHD symptoms via two distinct pathways: the altered “motivational style” pathway (MSP ADHD) which generates a strong aversion to experiencing delays, and the disordered “thought and action” pathway (DTAP ADHD) which results in a more fundamental dysregulation of inhibitory function. According to this dual-process model, those with DTAP ADHD may have a more generalised cognitive impairment, and exhibit qualitatively different behavior from normal children, whereas those with MSP ADHD only have difficulty in situations requiring the regulation of their behavior in time, and their symptomatology may reflect an extreme form of a normal personality trait. Moreover, it is suggested that these two forms of ADHD are underpinned by differing neurobiological mechanisms. DTAP ADHD may be primarily associated with dysregulation of prefrontal areas and their associated circuitry, particularly the connections with the basal ganglia and striatum. Such regions are regulated by the mesocortical dopaminergic system. In contrast, MSP ADHD may arise through alterations in the function of areas more fundamentally involved in reward processing, such as the ventral striatum, and innervated by the mesolimbic branch of the dopamine system (Sonuga-Barke, 2002).

This theoretical model has important implications not only for research into ADHD, but also for the understanding of impulsive behavior, and clearly supports the suggestion that different types of impulsivity are mediated by diverse neurobiological processes. It has recently been demonstrated in both humans and rats that different measures of impulsivity are not correlated within individuals (McDonald, Schleifer, Richards, & de Wit, 2003; Winstanley, Theobald, Dalley, & Robbins, 2004), suggesting that members of this collection of behaviors are independently regulated. The following section reviews some of the studies that have investigated which structures and neurotransmitter systems are involved in these different aspects of impulsivity, and speculates as to whether such data is compatible with a dual-process, or even multi-process, hypothesis of ADHD.

## 10 The neural basis of impulsive behavior

Frontostriatal systems have been heavily implicated in response inhibition, and dysfunction within this circuitry is thought to produce impulsivity associated with a number of psychological disorders (Jentsch & Taylor, 1999; Robbins, 2000). The roles of different areas of the frontal cortex and ventral striatum in various types of impulse control are considered below, and the preclinical data summarised in Table 1.

## 11 The frontal cortex

The cause of ADHD is currently unknown but a widely accepted hypothesis is that ADHD stems from dysfunction within the prefrontal cortex (PFC) (Castellanos & Tannock, 2002). Abnormalities in more ventral regions of the frontal lobes, such as the orbitofrontal cortex, have also been observed in ADHD (Hesslinger et al., 2002; Itami & Uno, 2002). The involvement of dysfunction within the frontal cortex in ADHD is supported by data demonstrating similarities between patients with injuries to or diseases of the frontal cortex and clinical aspects of ADHD (Shue & Douglas, 1992), particularly in terms of attentional dysfunction (Wilkins, Shallice, & McCarthy, 1987) and distractibility (Chao and Knight, 1995; Woods and Knight, 1986). Attentional impairments have also been observed in rats and monkeys with prefrontal cortex damage e.g.(Arnsten, 1997, 1998; Chao & Knight, 1995; Muir, Everitt, & Robbins, 1996; Passetti, Chudasama, & Robbins, 2002; Woods & Knight, 1986). However, there is also evidence to suggest that ADHD is not solely a prefrontal disorder, but that subcortical structures within the basal ganglia may play an important role (Rieger et al., 2003; Sergeant, 2000). Data from functional imaging studies comparing ADHD children to healthy controls found atypical fronto-striatal activation during performance of a go/no-go task, suggesting that abnormalities within this loop may account for some of the deficits in impulse control observed (Vaidya et al., 1998).

Considering such data from ADHD patients, it is perhaps unsurprising that dysfunction within the frontal cortex appears to be involved in generating impulsive behavior. A substantial body of evidence now exists to demonstrate deficits in a variety of inhibitory processes following damage to the frontal cortex. Recent neuropsychological studies implicate the prefrontal cortex both in terms of preparing to act (Brass & Von Cramon, 2002; Sohn, Ursu, Anderson, Stenger, & Carter, 2000) and switching between response alternatives (Dove, Pollman, Schubert, Wiggins, & Von Cramon, 2000; Mecklinger, Von Cramon, Springer, & Matthes-von Cramon, 1999; Rogers et al., 1998), as well as inhibiting inappropriate responses during strategy tasks (Dove et al., 2000; Mecklinger et al., 1999; Rogers et al., 1998; Shallice & Burgess, 1991a,b). Through the use of region of interest analysis, it has been possible to determine that damage to a specific region of the PFC, namely the right inferior frontal gyrus, could account for deficits in stop-signal inhibition associated with frontal cortex damage (Aron et al., 2003). Furthermore, it has been reported that human patients with damage to ventromedial frontal cortex, incorporating the orbitofrontal cortex (OFC), exhibit maladaptive decision-making and aberrant social behavior which is often described as impulsive. These patients persist in making risky choices on the Iowa gambling task (Bechara, Damasio, Damasio, & Anderson, 1994; Bechara, Damasio, Damasio, & Lee, 1999) and increased betting in the presence of normal probability judgements (Manes et al., 2002).

In the rat, lesions of the anterior cingulate cortex increase premature responding on the 5CSRT, whereas lesions to prelimbic cortex (analogous to dorsal PFC) impair attentional performance on the same task (Muir et al., 1996). However, large lesions of the medial PFC have not so far been found to affect performance on a stop-task in rats (Eagle & Robbins, 2003b). In addition, lesions to neither anterior cingulate nor prelimbic cortex altered impulsive choice as measured by delay-discounting paradigms (Cardinal et al., 2001). However, more recently it has been observed that rats with lesions to the OFC choose less impulsively in this task i.e. they prefer the larger but more delayed reward (Winstanley et al., 2004). Although this appears initially counter-intuitive with regards to the human literature on the Iowa gambling task, both lesion results could stem from a “myopia for the future” (Damasio, 1994) i.e. the subject chooses the response associated with the large reward regardless of the consequences of that response (delay to reward, monetary loss etc.). Perseverative responding has also been observed following lesions to the OFC, as determined through use of both the 5CSRT (Chudasama et al., 2003) and reversal learning paradigms (Chudasama & Robbins, 2003; Schoenbaum, Nugent, Saddoris, & Setlow, 2002) indicating an inflexibility within cognitive processes that may also impact on the impulsive-type behavior seen following damage to this region.

## 12 The ventral striatum

Numerous studies have underlined the importance of the nucleus accumbens (NAC) in the regulation of reward-related behavior. The NAC can be divided into two anatomically distinct parts: the core and the shell (Voorn, Gerfen, & Groenewegen, 1989; Zaborsky et al., 1985), which differ in terms of their patterns of innervation (Berendse, Galisdegraaf, & Groenewegen, 1992; Groenewegen, Vermeulen-van der Zee, Kortschot, & Witter, 1987; Voorn et al., 1989; Zaborsky et al., 1985; Zahm & Brog, 1992) and function (Baldo, Sadeghian, Basso, & Kelley, 2002; Corbit, Muir, & Balleine, 2001; Maldonado-Irizarry & Kelley, 1995; Parkinson, Olmstead, Burns, Robbins, & Everitt, 1999; Parkinson, Robbins, & Everitt, 1999; Sellings & Clarke, 2003). The NAC is a key node in the limbic corticostriatal loop, a circuit that is heavily implicated in goal-directed behavior and the evaluation of emotional stimuli and events. The NAC receives information from areas of frontal cortex, the hippocampal system and amygdala, and projects to motor output structures such as the caudate putamen and (indirectly) the mediodorsal thalamus (Alexander, Crutcher, & DeLong, 1990; Haber, Fudge, & McFarland, 2000). Given this pattern of interconnectivity, it has been suggested that the NAC acts as a “limbic–motor interface”, providing an important route whereby the limbic system can influence behavior (Mogenson, Jones, & Yim, 1980).

Excitotoxic lesions of the NAC core increase the number of premature responses made on the 5CSRT (Christakou, Robbins, & Everitt, 2004), yet have no effect on the stop-task (Eagle & Robbins, 2003b). However, damage to the NAC core markedly increases impulsive choice as measured by a delay-discounting task (Cardinal et al., 2001). More recently it has been observed that excitotoxic lesions of the basolateral amygdala (BLA), which is strongly connected to the NAC, increase impulsive choice in a similar fashion, indicating that the BLA and NAC may interact in the regulation of impulsivity (Winstanley, Theobald, Cardinal, & Robbins, 2004). Furthermore, damage to the ventral striatum and septal regions in the rat has been reported to decrease conditioned suppression, and to result in excessive alcohol drinking and aggression, all of which may indicate an overall increase in impulsivity (Johansson & Hansen, 2000).

Although lesions of the NAC core had no effect on performance of a rodent stop-task (Eagle & Robbins, 2003b), lesions to the medial striatum in the rat, an area homologous to the caudate putamen in the human brain, produced marked deficits in stop-task performance (Eagle & Robbins, 2003a). Most notably, SSRTs were significantly slower following medial striatal lesions, but the mean go response latencies (mRT) also increased, as did the number of trials omitted. The medial striatum in rats is probably homologous to regions of the caudate nucleus in humans, which has been shown to be important in the control of response inhibition in children with ADHD (Rubia, 2002; Semrud-Clikeman et al., 2000; Vaidya et al., 1998) and which may be smaller or dysfunctional in ADHD patients (Filipek et al., 1997; Rubia, 2002; Rubia et al., 1999). A recent study in human subjects also showed impaired performance on the stop-task following basal ganglia lesions (Rieger et al., 2003), although it was not possible to localise these lesions specifically to the caudate nucleus.

Medial striatal lesions also increase the incidence of premature responding on the 5CSRT (Rogers, Baunez, Everitt, & Robbins, 2001), but the effects of medial striatal lesions on the delay-discounting task are as yet unknown. Whether disturbances in striatal function are responsible for many of the behavioral deficits seen in ADHD patients is unclear, may warrant further investigation. The only other structure within the basal ganglia whose role in impulsivity has been investigated is the subthalamic nucleus (STN). Lesions of the STN increase impulsive responding as assessed by the 5CSRT (Baunez & Robbins, 1997), yet recent data suggest that damage to this structure decreases impulsive choice in a delay-discounting paradigm (Winstanley, Baunez, Theobald, & Robbins, 2005).

## 13 Summary

These data broadly support the suggestion that behavioral disinhibition and delay-discounting differ in the degree to which various components of frontostriatal loops are implicated in their regulation. In particular, the dorsal prefrontal cortex does not appear to be involved in mediating impulsive choice, yet does have some role in regulating inhibitory processes. In contrast, there appears to be a pronounced role for the OFC and BLA in controlling impulsive choice. Other structures, however, such as the NAC and STN may be common to both circuits. In terms of the dual process model of ADHD, it could be argued that these data are in agreement with the hypothesis that dysfunctions in prefrontal cortex and striatal systems are involved in DTAP ADHD, whereas structures heavily implicated in reward-learning and the processing of affect such as the NAC, BLA and OFC have a more pronounced role in MSP ADHD. As yet, such a conclusion must remain tentative, but continuing evaluation of the effects of manipulating different regions of the CNS on performance of these different tests of impulsivity may enable resolution of this issue. It is important to note that different measures of motoric impulsivity are differentially affected by lesions to various brain regions, once again suggesting that impulsivity is non-unitary in nature.

## 14 The neurochemical basis of impulsive behavior

The following discussion will focus on the roles of the dopamine and serotonin systems in the control of impulsivity. The noradrenergic system has also been implicated in the genesis and treatment of ADHD, yet little is known about the contribution this neurotransmitter makes to the regulation of impulsive behavior. Lesions to the dorsal noradrenergic bundle have limited effects on performance of the 5CSRT, mainly affecting accuracy under certain conditions (Cador, Taylor, & Robbins, 1991; Carli et al., 1983) yet their effects have not been assessed in other impulsivity paradigms. However, given the pattern of data emerging suggesting marked dissociations between the neural (see above) and neurochemical (see below) regulation of these different forms of behavior, further investigation of the noradrenergic system in preclinical studies of impulse control may prove useful.

## 15 The dopamine system

The most common pharmacological treatment prescribed for ADHD is the administration of amphetamine or methylphenidate, implicating dysregulation of the monoaminergic neurotransmitters, particularly dopamine or noradrenaline, in this disorder. It is seemingly paradoxical that psychostimulant drugs which increase activity should ameliorate the symptoms of ADHD, but this may be related to the rate-dependent nature of some of amphetamine's effects (Robbins & Evenden, 1985; Robbins & Sahakian, 1979). In support of this suggestion, amphetamine improved performance of the stop-task in both rodents and humans, but only in subjects with relatively poor baseline inhibitory performance, including those with medial striatal lesions (de Wit, Crean, & Richards, 2000; Eagle & Robbins, 2003a,b; Feola et al., 2000). Administration of d-amphetamine has also been shown to decrease impulsive choice in healthy human volunteers as assessed using a delay-discounting procedure (de Wit, Enggasser, & Richards, 2002), and produces a similar effect in rodent delay-discounting paradigms in the majority of studies (Isles, Humby, & Wilkinson, 2003; Richards, Sabol, & de Wit, 1999; Wade, de Wit, & Richards, 2000; Winstanley, Theobald, Dalley, & Robbins, 2003) but see also (Cardinal, Robbins, & Everitt, 2000; Evenden & Ryan, 1996).

Hence, there appears to be a high concordance between the behavioral effects of amphetamine on impulsive behavior in both humans and rodents, at least as measured by these two behavioral paradigms. In contrast, amphetamine increases the number of premature responses made on the 5CSRT, indicative of increased rather than decreased impulsivity (Cole & Robbins, 1987). This increase in impulsive responding is blocked by 6-OHDA lesions of the NAC, strongly implicating the involvement of accumbal dopamine in the actions of the psychostimulant (Cole & Robbins, 1989). Furthermore, the amphetamine-induced increase in the level of premature responding is blocked by administration of the D_1_ receptor antagonist SCH 23390, and also by global decreases in 5-HT (Harrison, Everitt, & Robbins, 1997), (see below for discussion of the role of the 5-HT system in impulsivity). The reasons why amphetamine appears to increase this measure of motoric impulsivity, yet decrease impulsive responding as measured by the stop-task, are currently unknown, but again indicate that different forms of impulsive action may have different neural bases.

The proposal that dysfunction of the dopamine (DA) system is responsible for some of the symptoms of ADHD is supported by reports of alterations in the dopamine transporter (DAT) (Dougherty et al., 1999; Krause, Dresel, Krause, Kung, & Tatsch, 2000) and abnormalities in DOPA decarboxylase activity (an enzyme involved in presynaptic storage of DA) within the PFC (Ernst, Zametkin, Matochik, Jons, & Cohen, 1998) in ADHD patients. Studies also suggest an association between ADHD and the dopamine D_4_ receptor gene polymorphism (e.g. (LaHoste et al., 1996)), and allelic versions of the DAT gene (Cook et al., 1995). Furthermore some of the behavioral abnormalities observed in the spontaneously hypertensive rat (SHR), a putative animal model of ADHD, are responsive to treatment with amphetamine and methylphenidate, although the effects of these stimulants and other dopaminergic drugs appear to be blunted in the SHR e.g. (Sagvolden, 1992; van den Buuse & de Jong, 1989; Yang, Amini, Swann, & Dafny, 2003). Significant abnormalities have been observed in dopaminergic innervation within the SHR, particularly with respect to the NAC (Papa et al., 2002; Papa, Sergeant, & Sadile, 1997; Russell, de Villiers, Sagvolden, Lamm, & Taljaard, 1998). The fact that both the dopamine transporter knockout mouse (DAT KO) and rats with neonatal 6-hydroxydopamine (6-OHDA) lesions are also hyperactive, and are considered animal models of ADHD, supports the view that dopamine is integral to the ADHD syndrome (see Solanto, 1998, 2002 for review).

## 16 The serotonin system

The hypothesis that serotonin (5-HT) is critically involved in impulse control has been gathering momentum for over 20 years. Soubrié proposed that a common basis for a number of different behavioral effects associated with decreases in brain 5-HT levels was the disinhibition of behavior, which can be directly related to the construct of impulsivity (Soubrie, 1986). Furthermore, Linnoila and colleagues established a correlation between low levels of the 5-HT metabolite 5-hydroxyindoleacetic acid (5-HIAA) in the cerebrospinal fluid (CSF), and impulsive rather than non-impulsive aggression (Linnoila et al., 1983). Despite these data indicating a strong association between 5-HT and impulsivity, the extent to which the 5-HT system is involved in ADHD is open to debate. Although low levels of 5-HIAA were observed in the CSF of a group of children and adolescents with disruptive behavioral disorders, including ADHD, this was found to correlate with levels of aggression rather than impulsivity per se, and could reflect the more general role of 5-HT in aggressive behavior (see Miczek, Fish, de Bold, & de Almeida, 2002 for review). However, a trend has also been observed between low levels of 5-HT in the blood and the severity of ADHD symptoms (Spivak et al., 1999), indicating that this neurotransmitter could somehow be involved in the behavioral disturbances central to this disorder.

In parallel with the dopamine hypothesis, genetic studies indicate an association between ADHD and a polymorphism in the 5-HT transporter gene (Retz, Thome, Blocher, Baader, & Rosler, 2002), and an allelic variant of the gene encoding the 5-HT_1B_ receptor (Quist et al., 2003). Nevertheless, the finding that serotonin-specific re-uptake inhibitors (SSRIs), which increase central 5-HT levels, have little or no effect in ADHD suggests that 5-HT may not be central to the aetiology of the disorder. However, in terms of other impulse-control disorders such as pathological gambling, sexual addiction and personality disorders, SSRIs have proved to offer some therapeutic benefit (Hollander & Rosen, 2000).

The effects of acutely reducing levels of 5-HT in the brain through tryptophan depletion on tests of impulsivity and behavioral disinhibition have been investigated in healthy volunteers as well as clinical populations and those with a history of psychiatric disorders. In general tryptophan depletion has been found to increase impulsive responding, particularly in those with a family history of psychiatric disorders, as measured by tests of impulsive action such as the stop-task procedure, go/no-go tasks and the CPT (Crean, Richards, & de Wit, 2002; LeMarquand, Benkelfat, Pihl, Palmour, & Young, 1999; LeMarquand et al., 1998; Quintin et al., 2001; Walderhaug et al., 2002), yet decreasing CNS 5-HT in this way has no effect in tests of impulsive choice such as delay-discounting and a simple test of probabilistic choice (Anderson, Richell, & Bradshaw, 2003). However, tryptophan depletion impairs reversal learning in an attentional set shifting task (Rogers et al., 1999) and subtly alters performance on a gambling test such that subjects did not change as much as controls in response to the size of the reward they could earn (Rogers et al., 2003). These data suggest that decreasing 5-HT does not necessarily increase impulsive choice per se, but does alter decision-making based on changes in the value of reward and rewarding stimuli.

In the rat, the hypothesis that decreasing 5-HT increases impulsive action has received widespread support. For example, globally reducing forebrain 5-HT through intracerebroventricular (ICV) infusions of the serotonergic toxin 5,7-dihydroxytryptamine, has been shown to increase premature responding on the 5CSRT and profoundly disrupted acquisition and performance of a go/no-go task (Harrison et al., 1997, 1999). However, whether decreasing 5-HT likewise increases impulsive choice in rats is less clear, as lesions to the serotonergic system have been reported to both increase impulsivity and to have no effect on performance of a delay-discounting paradigm (Bizot, Le Bihan, Puech, Hamon, & Thiebot, 1999; Mobini et al., 2000; Winstanley et al., 2003, 2004; Wogar et al., 1993). Although the reasons for these discrepancies are unclear, there are a number of obvious differences between these studies, not least the use of different behavioral tasks, varying methodology, and the presence or absence of neuroprotective pre-treatment strategies prior to surgery.

However, not all studies have found an inverse relationship between 5-HT levels and impulsive action. Focusing on the 5CSRT, a recent in vivo microdialysis study using a simplified “one-choice” version of the task found that, although levels of 5-HT did not alter during performance of the task, animals that made more premature responses on the task had *higher* levels of 5-HT in the medial PFC (Dalley, Theobald, Eagle, Passetti, & Robbins, 2002). A positive correlation has also been observed between levels of 5-HT in the right medial PFC and levels of premature responses made on the 5CSRT according to post mortem tissue analysis of trained animals (Puumala & Sirvio, 1998). It is important to remember that the 5-HT system is highly complex, and over 14 different types of 5-HT receptor have currently been identified which can have both excitatory and inhibitory effects on serotonergic neurons as well as the cells they target, including dopaminergic and glutamatergic neurons. Recent evidence suggests that drugs selective for these different 5-HT receptors can have radically different effects on impulsive behavior (Evenden & Ryan, 1999). In particular, whereas antagonism of the 5-HT_2C_ receptor antagonist SB 242084 increases premature responding, the 5-HT_2A_ receptor antagonist M100907 decreases this measure of impulsivity (Higgins, Enderlin, Haman, & Fletcher, 2003; Winstanley et al., 2003). Hence, antagonism of certain 5-HT receptors can decrease impulsive responding whereas global 5-HT depletion increases impulsivity on the same behavioral measure.

The essential diversity of the 5-HT system may provide an explanation for the inconsistencies in the literature regarding the role of this neurotransmitter in ADHD and impulsivity. It is clearly oversimplistic to view the serotonergic system as a homogeneous substrate upon which pharmacological agents act to produce monotonic changes in behavior. The finding that different serotonergic manipulations can have contrasting effects on different measures of impulsivity is an important consideration when evaluating the effects of serotonergic drugs in ADHD.

## 17 Interaction between 5-HT and DA

There is considerable evidence indicating that 5-HT can modulate levels of DA, and it has been suggested that changes in the *relative* levels of these two neurotransmitters, thus altering the balance of the interactions between them, could be one of the crucial factors in the neurochemical basis of ADHD (Oades, 2002). Amphetamine does increase levels of 5-HT as well as dopamine and noradrenaline (Balcioglu, Zhang, & Tarazi, 2003; Kuczenski & Segal, 1989, 1995; Kuczenski, Segal, Leith, & Applegate, 1987), therefore the therapeutic benefit derived from administration of amphetamine in ADHD may result in part from activation of the serotonergic system. In support of this suggestion, the hyperactivity observed in the DAT KO, a suggested rodent model of ADHD (see above), could be reduced by the 5-HT releasing agent fenfluramine, an effect which was abolished by lesions of the serotonergic system (Gainetdinov et al., 1999). Hyperactivity in rats with neonatal 6-OHDA lesions was also reduced by SSRIs, but not by inhibitors of the DAT (Davids, Zhang, Kula, Tarazi, & Baldesserini, 2002).

Direct evidence for the involvement of 5-HT:DA interactions in the control of impulsive choice in rats has been recently published. Although lesions to the 5-HT system did not themselves alter delay-discounting performance, such serotonergic lesions attenuated the ability of amphetamine to decrease impulsive choice, particularly in rats with high baseline levels of impulsivity (Winstanley et al., 2003). In addition, co-administration of the mixed dopamine antagonist *cis*-z-flupenthixol with amphetamine did not alter the ability of the psychostimulant to decrease impulsive choice in sham-operated rats, but did block these effects of amphetamine in ICV 5,7-DHT lesioned animals. Although amphetamine has opposite effects on impulsivity as assessed by delay-discounting tasks and the 5CSRT, it is interesting to note that, in both paradigms, the behavioral effects of amphetamine are attenuated by ICV 5,7-DHT lesions (Harrison et al., 1997; Winstanley et al., 2003). The differential involvement of interactions between the 5-HT and DA systems in these distinct forms of impulsive behavior may relate to the suggestion that DTAP ADHD and MPS ADHD are partly the results of dysfunction within different branches of the dopaminergic system, although such a suggestion remains to be tested.

## 18 Summary

The neurotransmitter most clearly implicated in ADHD is dopamine, and data from both clinical and preclinical studies largely support this suggestion. Although there is a considerable body of literature associating the serotonin system with impulsivity, the precise role this system plays, if any, in the etiology and treatment of ADHD is yet to be determined. Preclinical evidence is mounting to suggest that 5-HT may be important in the action of amphetamine to reduce impulsive behavior, potentially via its interactions with the dopamine system. Whilst the nature of such an interaction is still unclear, further investigation of this issue may provide valuable insight into the neurochemical basis of ADHD.

## 19 Discussion

The body of data reviewed here highlights the advances that have been made in understanding the neurobiological basis of impulsivity through empirical investigation of behavior within the laboratory setting. Through designing behavioral paradigms for use with rodents and other laboratory animals based on human neuropsychological tests, it has proved possible to replicate findings between species, and to validate the utility of such a behavioral test strategy to investigate impulsive behavior. Such experiments have highlighted the role of frontostriatal circuits in impulse control in both humans and rodents e.g. (Aron et al., 2003; Bechara et al., Lee, 1999; Cardinal, Pennicott, Sugathapala, Robbins, & Everitt, 2001; Christakou et al., 2004; Muir et al., 1996; Winstanley et al., 2004), but also suggest that different, yet converging, pathways may regulate distinct aspects of impulsive behavior. This *fractionation* of impulsivity, both in terms of behavioral output, neural basis and neurochemical modulation, may illuminate some issues regarding the nature of ADHD. In particular, this literature supports the suggestion that more than one subtype of ADHD may exist, characterised by similar symptoms that may differ in severity, yet which are caused by dysfunction within different brain systems.

Further work to delineate the precise roles of different regions of the brain in the control of different forms of impulsive behavior clearly remains to be done in order to establish a more complete picture of the areas and circuits involved in various forms of impulsivity. The identification of the common as well as the unique involvement of different areas may further our understanding of the complex pattern of data gathered from populations of patients with impulse control disorders, and predict and support hypotheses regarding therapeutic strategies and origins of such syndromes. Appreciation of the complexity of the different neurotransmitter systems implicated in impulsivity and ADHD, and the interactions between them, may lead to further advances in pharmacological control of impulsivity. In particular, preclinical studies indicate that drugs that are selective for particular receptor subtypes may have distinct and more pronounced effects than globally acting drugs. Future work may establish such agents as useful adjuncts to more traditional pharmacotherapy for impulse control disorders.

To conclude, both clinical and preclinical studies have made valuable contributions to our understanding of impulsivity and its involvement in ADHD. The considerable translation possible between the behavioral models in both rats and humans has enriched and advanced our comprehension of the neurobiological basis of these behaviors. Continuing communication and exchange of ideas between neuroscientists and neuropsychologists working in these fields can only be beneficial in advancing progress in this area.

## Figures and Tables

**Table 1:** Simple summary of the effects of lesions to different brain regions on various tests of impulsivity in the rat

Region	Stop-task	Delay-discounting	5CSRT
PRL	↔	↔	↔
IL	?	↔	↑
ACx	?	↔	↑
OFC	?	↓	↑
NAC	↔	↑	↑
Medial striatum	↑	?	↑
STN	?	↓	↑

↔: no effect on performance; ↑: increase in impulsive behavior; ↓: decrease in impulsive behavior; ?: effects unknown. Abbreviations: 5CSRT five-choice serial reaction time task; PrL prelimbic cortex; IL infralimbic cortex; ACx anterior cingulate cortex; OFC orbitofrontal cortex; NAC nucleus accumbens; STN subthalamic nucleus.
